# Structural Differences in Gray Matter between Glider Pilots and Non-Pilots. A Voxel-Based Morphometry Study

**DOI:** 10.3389/fneur.2014.00248

**Published:** 2014-11-28

**Authors:** Tosif Ahamed, Motoaki Kawanabe, Shin Ishii, Daniel E. Callan

**Affiliations:** ^1^Cognitive Mechanisms Laboratories, Advanced Telecommunications Research Institute International (ATR), Kyoto, Japan; ^2^Okinawa Institute of Science and Technology, Okinawa, Japan; ^3^Kyoto University, Kyoto, Japan; ^4^Center for Information and Neural Networks (CiNet), National Institute of Information and Communications Technology (NICT), Osaka University, Osaka, Japan; ^5^Multisensory Cognition and Computation Laboratory, Universal Communication Research Institute, National Institute of Information and Communications Technology, Kyoto, Japan

**Keywords:** voxel-based morphometry (VBM), experience-dependent plasticity, anterior cingulate cortex (ACC, RCZa), ventral premotor cortex (PMv), supplementary eye field, vestibulo-ocular reflex, vestibular habituation

## Abstract

Glider flying is a unique skill that requires pilots to control an aircraft at high speeds in three dimensions and amidst frequent full-body rotations. In the present study, we investigated the neural correlates of flying a glider using voxel-based morphometry. The comparison between gray matter densities of 15 glider pilots and a control group of 15 non-pilots exhibited significant gray matter density increases in left ventral premotor cortex, anterior cingulate cortex, and the supplementary eye field. We posit that the identified regions might be associated with cognitive and motor processes related to flying, such as joystick control, visuo-vestibular interaction, and oculomotor control.

## Introduction

In order to keep up with the demands of a changing environment, our brains adapt quickly and efficiently. This is best demonstrated by gray and white matter structure changes in brain regions associated with practicing specific motor or cognitive skills. Such findings have been reported by many cross-sectional studies done over the past decade comparing trained experts to non-experts. Now termed as experience-dependent structural plasticity, the process is thought to be active throughout our lives (1, 2). For instance, various voxel-based morphometry (VBM) studies comparing brains of musicians to non-musicians found increased gray matter density (GMD) in several brain regions in musicians, including the cerebellum, auditory, and motor cortices (3, 4). Skilled golfers were reported to have larger GMD in premotor and parietal areas (5). Long-term practice might also result in a decrease of gray matter as was the case with ballet dancers where authors reported decreased gray matter volumes in the left premotor cortex, supplementary motor area, putamen, and superior frontal gyrus (6). Structural changes can also be observed in purely cognitive skills, e.g., mathematicians were shown to have larger inferior frontal and bilateral inferior parietal lobules (7). Bilinguals had larger GMD in the inferior parietal cortex compared with monolinguals (8).

Flying a glider is a unique skill as human beings are not naturally suited for operation in a non-terrestrial environment. Very little is known about how the brain of a glider pilot adapts to the needs of being in the air, which are very different from other land-based motor skills. Glider flying involves operation in three dimensions, at considerably variable velocities, altitudes, and g-forces. To avoid motion sickness, pilots must habituate to the unusual visual–vestibular interaction resulting from full-body rotation within a thermal column. Glider pilots have to simultaneously integrate multiple streams of sensory information from visual, vestibular, and kinesthetic systems to form a mental construct of their position and orientation to control the glider. Specifically, pilots use a joystick with one hand to control the roll and pitch of the glider, foot pedals to control the yaw, and a dive brake with the other hand to increase the drag during landing. Co-ordination of all four degrees of freedom is required to be able to fly and land a glider properly. Apart from precise sensorimotor control, flying demands high levels of cognitive control, as pilots have to continuously monitor their performance based on multimodal sensory feedback mechanisms. Moreover, the process has to be predictive, has a low margin of error, and often pilots have to resolve conflicting information coming in from different senses. These factors make flying a very interesting skill to study from a neuroscience perspective and investigating the neural correlates of flying has the potential to throw light on many brain processes involved in motor control, multisensory integration, and cognitive control.

Recently, a few studies from our group reported functional activation patterns as subjects tried to fly an aircraft in a flight simulator inside a MRI scanner (9, 10). Despite the significance of the findings, the utility of this method in revealing the underlying neural basis of flying as a sensorimotor skill is limited by the space and movement restrictions of fMRI. Looking at structural differences between the brains of pilots and non-pilots presents us with a viable alternative.

Previous studies that have looked at physiological differences between pilots and non-pilots, point toward vestibular habituation and adaptation of the vestibulo-ocular reflex (VOR) in pilots (11–14). VOR is an eye reflex that moves the eye in a direction opposite to the head movement. VOR adaptation in pilots suggests that the mechanism is important for stabilizing images on the retina during head and full-body rotations as the glider rotates in a thermal column. Psychophysical tests have shown that fighter pilots have superior cognitive control as compared to non-pilots as measured by the Eriksen Flanker task (15). The same study also found differences in white matter radial diffusivity (derived from diffusion weighted imaging) between fighter pilots and non-pilots in the right dorsomedial frontal region and parietal lobe. These studies predict that compared to non-pilots, pilots may have changes in GMD in brain regions related to vestibular habituation, motor learning, sensorimotor integration, and cognitive control. Additionally, the results of the flight simulator fMRI studies (9, 10) suggest that these brain regions include but are not limited to the ventral premotor cortices, inferior parietal lobule, supplementary motor area, and few areas in occipital and temporal lobe.

In the present study, we wanted to investigate the structural correlates of flying a glider by analyzing gray matter differences between glider pilots and non-pilots using VBM. Unlike the previous study done to detect changes in of white matter structure between fighter pilots and non-pilots (15), we looked at gray matter and did not use any masks to restrict our search.

## Materials and Methods

### Ethics statement

All subjects gave written informed consent for experimental procedures approved by the ATR Human Subject Review Committee in accordance with the principles expressed in the Declaration of Helsinki.

### Subjects

Thirty right-handed subjects participated in this study. The handedness of the subjects was determined using a questionnaire based on Edinburgh Handedness Inventory (16). Fifteen of the subjects were glider pilots recruited from nearby gliding clubs. The pilots were all well experienced with a mean in-air flight experience of 34.2057 h (SE 5.35), where an average glider flight lasts 10–15 min. All pilots reported using their right hand to control the joystick. All subjects in the control non-pilot group had experience with driving or flying related video games. Age and sex between the two groups was balanced. Pilots had a mean age of 21.3 years (SE = 0.36), while the control non-pilot group had a mean age of 22.4 years (SE = 0.49). Age effects were also controlled for by including age as a confounding regressor in the statistical model. There were 13 males and 2 females in both the pilot and control groups. All subjects were Japanese, came from similar educational and socioeconomic backgrounds, and had no history of neurological, head trauma, or psychiatric disorders.

### Image acquisition

High-resolution anatomical scans were acquired with T1 weighting (TE = 3.06 ms, TR = 2.25 s, matrix size = 256 × 256, voxel size = 1 mm × 1 mm × 1 mm) were acquired on a Siemens Trio 3 T scanner at the ATR Brain Activity Imaging Center.

### Voxel-based morphometry analysis

Voxel-based morphometry is a method used to automatically analyze differences in local brain anatomy (17). T1 weighted structural MR images were used as an input to the VBM pipeline. All T1 images were processed using SPM8 (Wellcome Department of Cognitive Neurology, UCL), running under MATLAB 7.13 on a Linux platform (The Mathworks, Natick, MA, USA). VBM was performed using the VBM extension present in SPM8. The preprocessing involved following steps:

1. After checking raw images for artifacts and setting the origin to Anterior Commissure (AC), they were segmented into GM, WM, and CSF using unified segmentation (18) (New Segment toolbox in SPM8).
2. All images were then warped to a study specific template created using the DARTEL registration algorithm (19) in SPM8.
3. To preserve the original GM volume, flow fields generated by DARTEL in the previous step were combined to generate Jacobian scaled GM images.
4. Warped and Jacobian scaled images were transformed to Montreal Neurological Institute (MNI) space and smoothed by a Gaussian kernel of 8 mm FWHM. The smoothed images were then used for statistical analysis.

General linear model as implemented in SPM8 was used for all statistical analysis. Differences in GMD between the two groups were analyzed using one-way ANCOVA. Data were corrected for global brain volume by dividing each voxel by the total intracranial volume and age was added as a regressor of no interest. Voxelwise statistical parametric maps showing differences in GMD between pilot and non-pilot groups were generated by setting the voxel level threshold at *t* > 4.94, *p* < 0.05 [corrected for multiple comparisons using false discovery rate (FDR)]. The initial localization of brain regions that were found significant was done using SPM Anatomy toolbox (20) localization was further refined based on anatomical parcelation literature as mentioned in the discussion below.

## Results

**Figure 1:**
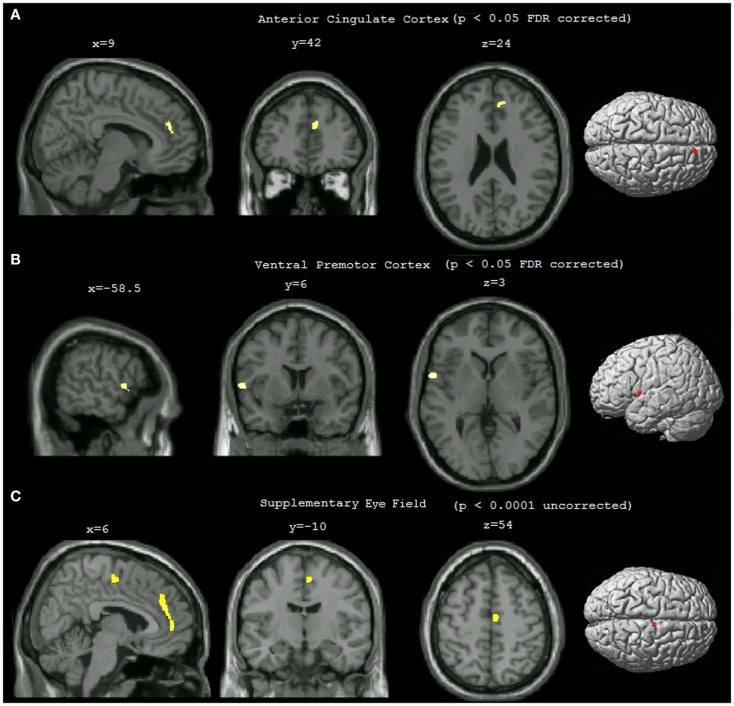
**Statistical parametric maps showing differences in gray matter density between pilots and non-pilots**. The maps were overlaid on the MNI aligned 3D image of a selected single subject and rendered on the average brain surface provided in SPM8. **(A)** Right anterior cingulate cluster (*p* < 0.05 FDR corrected, cluster threshold = 25). **(B)** Left ventral premotor cortex cluster (*p* < 0.05 FDR corrected, cluster threshold = 25). **(C)** Right supplementary eye field (*p* < 0.0001 uncorrected, cluster threshold = 25).

Statistical analysis showed that compared to non-pilots, pilots had significantly higher GMD in the left ventral premotor area (lPMv) and right anterior cingulate cortex (rACC) (Table 1; Figures 1A,B), *p* < 0.05 FDR corrected for multiple comparisons. Lowering the threshold to *p* < 0.0001 uncorrected, showed another cluster in the right supplementary eye field (rSEF) within the supplementary motor area, where pilots had a higher GMD compared to the non-pilot group (Table 1; Figure 1C). No regions were found to have significantly lower GMD in pilots (uncorrected *p* < 0.0001).

**Table 1:** **Regions of GMD differences between pilots and non-pilots obtained by VBM analysis. Co-ordinates for peak voxels are in MNI space**.

Area	Number of voxels	Peak co-ordinate (*x*,*y*,*z*)	Brodmann area
FDR corrected (*p* < 0.05)
Left PMCv	63	−58.5, 6, 3	6
Right ACC	101	9, 42, 24	9
Uncorrected (*p* < 0.0001)
Right SEF (SEF)	71	6, −10, 54	6

*PMCv, ventral premotor cortex; ACC, anterior cingulate cortex; SEF, supplementary eye field*.

Individual GMD values within the pilot group extracted from peak voxels of the two significant clusters showed no significant correlation (*p* > 0.05) with the number of hours of in-air flight experience.

## Discussion

To the best of our knowledge, our study is the first to demonstrate structural differences in the gray matter of glider pilots. We show that pilots have increased GMD in regions that can all be grouped under the premotor areas of the frontal lobe, regions that influence various kinds of motor output through projections to the primary cortex and spinal cord (21). Because of the complexity of the skill and paucity of previous work on the neuroanatomical correlates of flying, it is difficult to say precisely what role these brain regions play in this particular skill. However, based on a literature review, plausible interpretations for their involvement are discussed below; the interpretations are speculative but informative.

### Ventral premotor cortex

As per a recent parcelation of the lateral premotor cortex, our lPMv blob lies in the cluster corresponding to area F5 in macaque (22). In the literature, this area has been repeatedly shown to be involved in grasping and manipulation of objects, as well as conditional motor learning (23, 24). Learning-dependent activity has been shown to occur in this region as subjects acquire new visuomotor associations to manipulate a joystick (25). In the aforementioned VBM study, golfers (who have to learn precise visuomotor control of golf clubs) (5) were found to have higher GMD in this same region. Functional activation patterns were also observed in this region in the flight simulator studies and are thought to underlie visuomotor processes encompassing the mirror neuron system involved with movement imitation and imitation learning (9, 10). All these sources of evidence point toward the involvement of the ventral premotor cortex in acquisition of new visuomotor associations as a pilot learns to control the glider using a joystick. The left lateralization of this cluster can be explained by the fact that all subjects being right handed were used to manipulating the joysticks with their right hand.

### Anterior cingulate cortex

The rACC cluster is located in the anterior rostral cingulate zone (RCZa) (21). According to most studies, this region of the cortex is said to be involved in conflict monitoring and motor related cognitive control (26, 27). Often the different senses involved in flying send conflicting information to the brain, for instance when a glider is soaring, the visual system might give an impression of stillness, while the vestibular system senses self-motion. Thus, conflict monitoring and decision making in conflict is an important aspect of flying. Structural reorganization of rACC as the skill develops is in accordance with the accepted function of this region. A more relevant involvement of ACC in flying comes from a study that reported increased activation of ACC with repeated vestibular stimulation, pointing toward the involvement of this region in adaptation of the VOR (28). As mentioned previously, VOR adaptation has been reported in pilots and can even be used to differentiate pilots from non-pilots (11, 14). Several other studies have reported significant activations of ACC in vestibular stimulation and visual–vestibular interaction experiments (29). This interpretation is strengthened by the fact that the flight simulator studies, which had no vestibular component reported no functional activation in this region.

### Supplementary eye field

The cluster found in the supplementary motor area can be localized to a specialized region called the supplementary eye field (SEF) (30). Across human and monkey studies, this region has been reported to be involved in various aspects of oculomotor control, such as learning oculomotor transformations, smooth pursuit, and cognitive control of the oculomotor system like performance monitoring and prediction (31, 32). Amidst all the head and full-body rotation involved in flying, pilots require a fine-tuned oculomotor system to control their eye movements so that the visual image is stable on the retina. We believe that SEF is one of the areas that fulfill this role. SEF is also reported to be involved in suppression of nystagmus, which in turn is related to vestibular habituation in pilots (12, 13). Further support to this interpretation comes from the fact that this area was also found active in previous flight simulation fMRI studies (9, 10). Thus, the increase in GMD in the SEF is probably involved with the abovementioned oculomotor functions crucial to flying.

Evidently, the brain regions found significant in the present study could be responsible for physiological and perceptual processes involved in flying, such as motor learning, vestibular habituation, and cognitive control. The lack of correlation between in-air flight experience and GMD of the brain structures found significant may have several reasons. The lack of a significant correlation may be explained by the fact that habituation is a fast process and by the time a pilot is good enough to fly a real glider on his own, his eyes and vestibular senses are already well habituated. An additional explanation may be that in-air flight experience in our study is not a sensitive measure of differences in individual skill. It may be the case that our sample size is not large enough to capture such small differences in skill-related experience that is thought to be reflected by greater GMD in specific cortical regions. It should be pointed out that the aforementioned study, which looked at trained fighter pilots also did not find any correlations between flying hours and white matter changes (15). To the best of our knowledge, the neural correlates of vestibular habituation are not very well known; accordingly one of the key insights of this study is the possible involvement of ACC and SEF in the process of vestibular habituation.

## Conclusion

The results of our study show that glider pilots have increased GMD in ventral premotor cortex, anterior cingulate cortex, and supplementary eye field, which are associated with sensorimotor learning, visual–vestibular interaction, and oculomotor control, respectively. Further studies are needed to evaluate the degree to which performance of flight-related tasks can be predicted from GMD in these regions and the longitudinal pattern of the changes.

## Conflict of Interest Statement

The authors declare that the research was conducted in the absence of any commercial or financial relationships that could be construed as a potential conflict of interest.
